# Aorto-Right Atrial Fistula Caused by Subacute Type A Aortic Dissection Six Months After Double Valve Replacement: A Case Report and Literature Review

**DOI:** 10.7759/cureus.86692

**Published:** 2025-06-24

**Authors:** Hiroto Yasumura, Goichi Yotsumoto, Yoshihiro Fukumoto, Yuki Ogata, Koichiro Shimoishi, Tomoyuki Matsuba, Kenichi Arata, Hideyuki Satozono, Yushi Yamashita, Yoshikazu Kawazu, Kenji Toyokawa, Hiroyuki Yamamoto, Yoshiharu Soga

**Affiliations:** 1 Department of Cardiovascular Surgery, Kagoshima City Hospital, Kagoshima, JPN; 2 Department of Cardiovascular Surgery, Graduate School of Medical and Dental Sciences, Kagoshima University, Kagoshima, JPN; 3 Department of Cardiovascular Surgery, Yonemori Hospital, Kagoshima, JPN

**Keywords:** aortic atrial fistula, aorto-right atrial fistula, dissection rupture, double valve replacement, type a aortic dissection

## Abstract

Type A aortic dissection (TAAD) in Stanford classification after previous cardiac surgery is a rare but serious complication, with an incidence of 0.1%-0.2%. Previous sites of cannulation, cross-clamping, and anastomosis of vein grafts have been reported as potential entry points for aortic dissection. In some cases, the aortic dissection ruptures into a neighboring atrial chamber due to dense postoperative adhesions. In this report, we present a rare case of TAAD with an aorto-right atrial fistula after double valve replacement (DVR) and provide a literature review.

A 76-year-old Japanese man presented with exertional dyspnea due to severe aortic regurgitation, moderate to severe mitral regurgitation, and paroxysmal atrial fibrillation. Preoperative plain computed tomography (CT) revealed ascending aorta and sinus of Valsalva diameters of 45 mm each. DVR and left atrial appendage (LAA) occlusion were performed. He was uneventfully discharged on postoperative day (POD) 21. Six months after the discharge, the patient complained of chest and back pain, orthopnea, and appetite loss. A family doctor firstly diagnosed him with muscle pain and belatedly realized that he suffered from acute heart failure and possible TAAD, leading to his referral to our hospital. Contrast-enhanced CT and echocardiography revealed an aorto-right atrial fistula caused by a subacute TAAD. Despite medical management, heart failure could not be controlled, necessitating emergency surgery. During the operation, the entry point of the TAAD was identified as a healthy aortic wall located just behind the previous aortotomy. An 8-mm fistula into the right atrium was observed from the false lumen. The fistula was closed with two 4-0 polypropylene felted mattress sutures only from the false lumen side and the ascending aorta was replaced with J-graft. Postoperative magnetic resonance imaging revealed a left pontine infarction, but all other postoperative examinations were unremarkable. The patient was transferred to another hospital on POD 43. Six months after the TAAD operation, he underwent 1-debranching thoracic endovascular aortic repair (TEVAR) for residual descending aortic dissection. He has been alive for five years since the TEVAR.

Successful closure of the fistula and replacement of the ascending aorta were achieved in a patient with aorto-right atrial fistula caused by subacute TAAD after DVR and LAA occlusion. When preoperative imaging shows an ascending-aorta diameter ≥45 mm, it may be reasonable to discuss the concomitant replacement of aortic root, ascending aorta and partial /hemi arch, and the cannulation strategies, in line with American College of Cardiology and the American Heart Association (ACC/AHA) Class IIa guidance. Given the paucity of AAF cases after valve surgery, further multicenter series or registry data are needed to validate the optimal diameter threshold and cannulation approaches for preventing postoperative dissection.

## Introduction

Type A aortic dissections (TAAD) in the Stanford classification involve the ascending aorta, and type B aortic dissections do not. Aortic dissection occurs in 10 of 100,000 people per year and 61.4% of the patients die before arrival in the hospital [1]. TAADs carry a mortality of 1% to 2% per hour in the first 24-48 hours if left untreated [2].

TAAD after previous cardiac surgery (PCS) is a rare but serious complication, with an incidence of 0.1%-0.2% [3-5], and therefore leads to only a few reports. Previous sites of cannulation, cross-clamping, and vein graft anastomosis have been reported as potential entry points for aortic dissection [6-8]. It is because those maneuvers may create focal intimal stress or weaken the medial layer.

In patients with TAAD without a history of PCS, the dissected aortic wall can rupture freely into the pericardial sac, mediastinum, or thoracic cavity, often resulting in sudden death. In contrast, in patients with TAAD after PCS, the dissected aortic wall and a pseudoaneurysm of the sinus of Valsalva may rupture into a neighboring right and left atrium due to dense postoperative adhesions [9,10]. Those communications are called aorto-atrial fistulas (AAFs) and can mitigate the otherwise fatal morbidity. The primary cause of AAFs is endocarditis (22.8%) of native or prosthetic valves. It was followed by ascending aortic aneurysms with dissection (16.2%) and without dissection (5.9%) [11].

In this report, we present a rare case of TAAD with an aorto-right atrial fistula after double valve replacement (DVR) along with a literature review, contributing to the early detection of TAAD after PCS.

## Case presentation

Initial presentation of valvular disease

A 76-year-old Japanese man (height 166.4 cm, weight 71.0 kg) with a history of spontaneous pneumothorax and cholecystitis complained of exertional dyspnea. He was referred to our hospital.

Diagnostic workup

**Figure 1:**
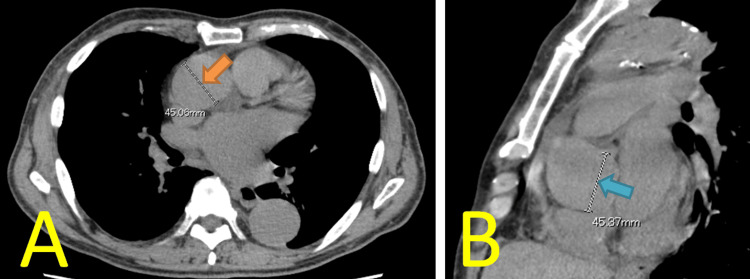
Preoperative findings of double valve replacement A. Plain computed tomography revealed that the ascending aorta was 45 mm in diameter (orange arrow, axial view). B. The sinus of Valsalva was 45 mm in diameter (blue arrow, sagittal view).

Echocardiography revealed severe aortic regurgitation (AR), moderate to severe mitral regurgitation (MR), and paroxysmal atrial fibrillation. The aortic valve had three leaflets, and the AR was caused by valve prolapse. The mitral valve was tethered, with the posterior leaflet immobile and the anterior leaflet relatively prolapsed (Carpentier classification Ⅲb), resulting in an eccentric MR jet. Preoperative plain computed tomography (CT) revealed that the diameters of the ascending aorta and sinus of Valsalva were 45 mm each (Figure 1A, 1B). Coronary angiography revealed no significant stenosis.

Operative findings

Under general anesthesia, cardiopulmonary bypass (CPB) was established using the ascending aorta, superior vena cava (SVC), and inferior vena cava. Double valve replacement (DVR) with St Jude Medical Epic bioprosthesis (St Jude Medical Inc., St Paul, MN, USA) and left atrial appendage (LAA) occlusion from inside the left atrium were performed without complications.

Postoperative course

The postoperative course was uneventful. Laboratory test on postoperative day (POD) 20 showed almost normal renal function, indicating blood urea nitrogen (BUN) at 21.2 mg/dL and creatinine at 1.02 mg/dL. He was discharged on POD 21.

Initial presentation of AAF due to type A aortic dissection

Six months after the discharge, the patient suddenly developed chest and back pain. He saw his family doctor. Electrocardiography revealed sinus bradycardia of 40 beats per minute and no significant ST elevation. Chest radiography showed the same enlarged mediastinum as the one in our hospital. He was diagnosed with muscle pain and followed with an additional analgesic and a decreased beta blocker. One week later, he complained orthopnea, which worsened when lying down, and loss of appetite. Five days later, he saw the doctor on foot again. Laboratory test showed brain natriuretic peptide was 1066 pg/mL (reference value: <18.4 pg/mL) and chest radiography showed a new right dominant pleural effusion. He was diagnosed with acute heart failure and was admitted to the hospital. Intravenous furosemide improved orthopnea. On the third hospital day, a plain CT, considering renal failure, suggested type A aortic dissection (TAAD). The patient was referred to our hospital.

Diagnostic workup

Physical examination revealed facial edema, jugular vein distention, and a prominent pan-systolic heart murmur at the second right sternal border. The patient’s weight had increased to 79.2 kg. Laboratory test on admission to our hospital showed white blood cell count at 10.3×103/μL, hemoglobin at 11.4 g /dL, platelet count at 31.3×103/μL, aspartate aminotransferase (AST) at 85 U/L, alanine aminotransferase (ALT) at 156 U/L , alkaline phosphatase (ALP) at 394 U/L, lactate dehydrogenase (LDH) at 364 U/L, γ-glutamyl transpeptidase (γGTP) at 71 U/L, BUN at 89.9 mg/dL, creatinine at 2.16 mg/dL, and C-reactive protein (CRP) at 3.08 mg/dL (Table 1). Blood culture results were negative.

**Table 1:** Laboratory investigation results on admission to our hospital This table includes key hematological parameters with elevated values in bold. The elevated white blood cell and C-reactive protein reflect the inflammation due to aortic dissection. The elevated liver enzymes, BUN and creatinine reflect the congestion of liver and kidney due to aorto-right atrial fistula caused by aortic dissection.

Parameter	Obtained Value	Reference Range
White Blood Cell Count	10.3×10^3^/μL	3.3-8.6×10^3^/μL
Hemoglobin	11.4 g/dL	13.7-16.8 g/dL
Platelet Count	313×10^3^/μL	158-348×10^3^/μL
Aspartate Aminotransferase (AST)	85 U/L	13-30 U/L
Alanine Aminotransferase (ALT)	156 U/L	10-42 U/L
Alkaline Phosphatase (ALP)	394 U/L	106-322 U/L
Lactate Dehydrogenase (LDH)	364 U/L	124-222 U/L
γ-Glutamyl Transpeptidase (γGTP)	71 U/L	13-64 U/L
Blood Urea Nitrogen (BUN)	89.9 mg/dL	8.0-20.0 mg/dL
Creatinine	2.16 mg/dL	0.65-1.07 mg/dL
C-Reactive Protein (CRP)	3.08 mg/dL	0.00-0.14 mg/dL

**Figure 2:**
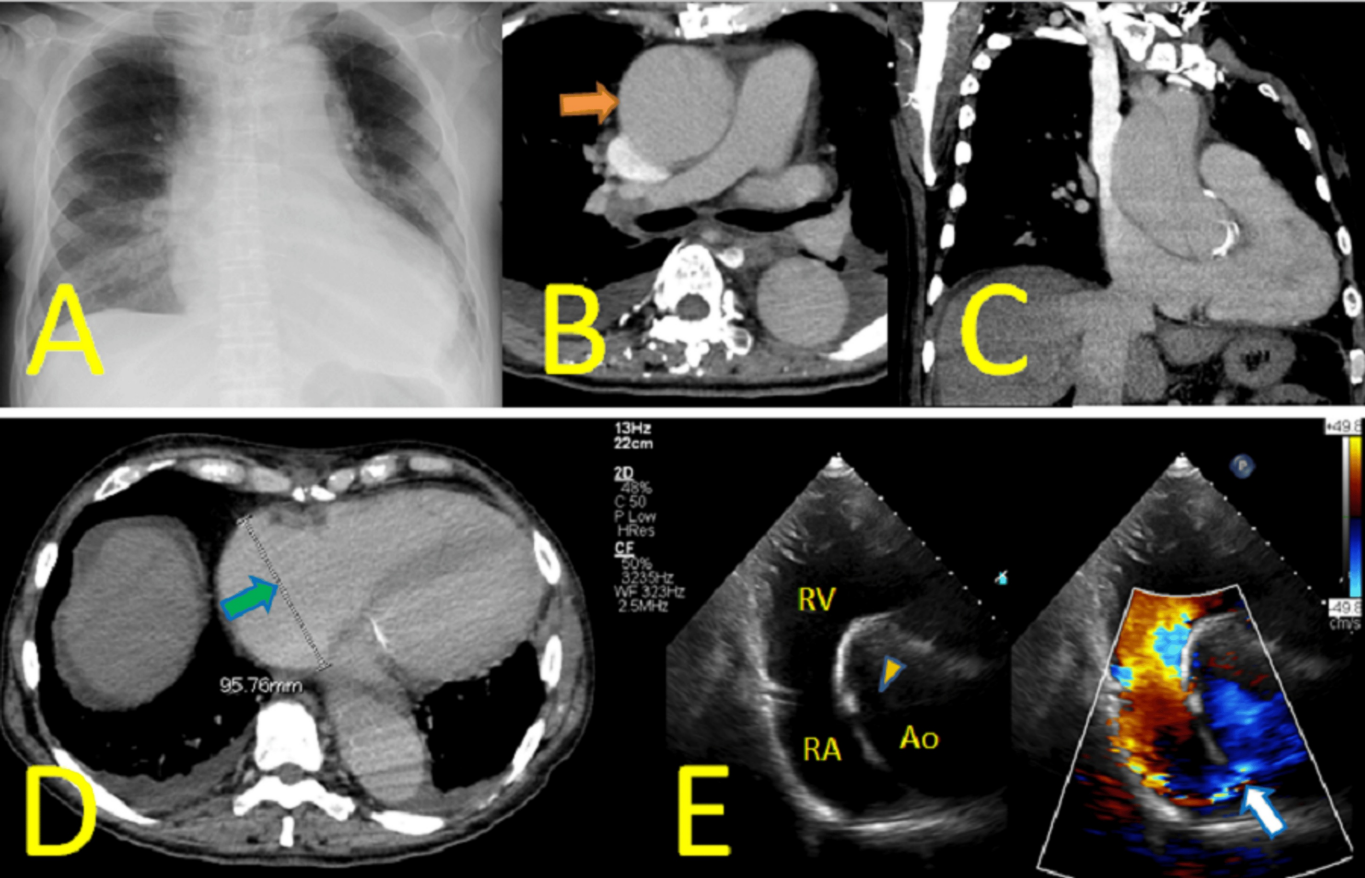
Preoperative findings of type A aortic dissection A: Chest radiography revealed cardiac and mediastinal enlargement. B: Contrast-enhanced CT showed that the dissected ascending aorta had enlarged to 60 mm in diameter (orange arrow). C: The reduced use of contrast agent made it difficult to identify the dissection entry point. D: The right atrium had enlarged to 96 mm (green arrow). E: Transthoracic echocardiography revealed a shunt flow (white arrow) from the false lumen of the ascending aorta (Ao) to right atrium (RA). The yellow arrowhead indicates the medial flap. RV is right ventricle.

Chest radiography revealed cardiac and mediastinal enlargement (Figure 2A). Contrast-enhanced CT showed that the ascending aorta, which was 45mm in diameter eight months ago, enlarged to 60 mm due to aortic dissection (Figure 2B). The dissection extended from the aortic root to the descending aorta, indicating DeBakey classification type I. The brachiocephalic artery was dissected, but the false lumen had persistent perfusion. Left common carotid artery and left subclavian artery were perfused from the true lumen. Carotid ultrasonography revealed no malperfusion among those three main branches of the aortic arch. The reduced dose of contrast agent considering renal failure made it difficult to identify the dissection entry point (Figure 2B, 2C). Additionally, the right atrium had enlarged to 96 mm (Figure 2D). The first echocardiography on admission day showed neither AR, pericardial effusion, nor shunt flow, but repeated echocardiography on the third and fifth hospital day identified a shunt flow from the false lumen of the ascending aorta to the right atrium. The shunt flow was located 7 cm distal to the aortic valve annulus (Figure 2E). This finding confirmed an aorto-right atrial fistula caused by subacute TAAD. The shunt flow became increasingly detectable and showed progression with repeated echocardiography. Initially, intravenous diuretics provided symptomatic relief. Considering the TAAD was subacute phase, an elective surgery was planned. However, worsening biventricular heart failure due to increased shunt flow necessitated ultrafiltration with continuous hemodiafiltration (CHDF), which became difficult to improve pulmonary edema. Therefore, emergency surgery was performed on the sixth hospital day.

Operative findings

**Figure 3:**
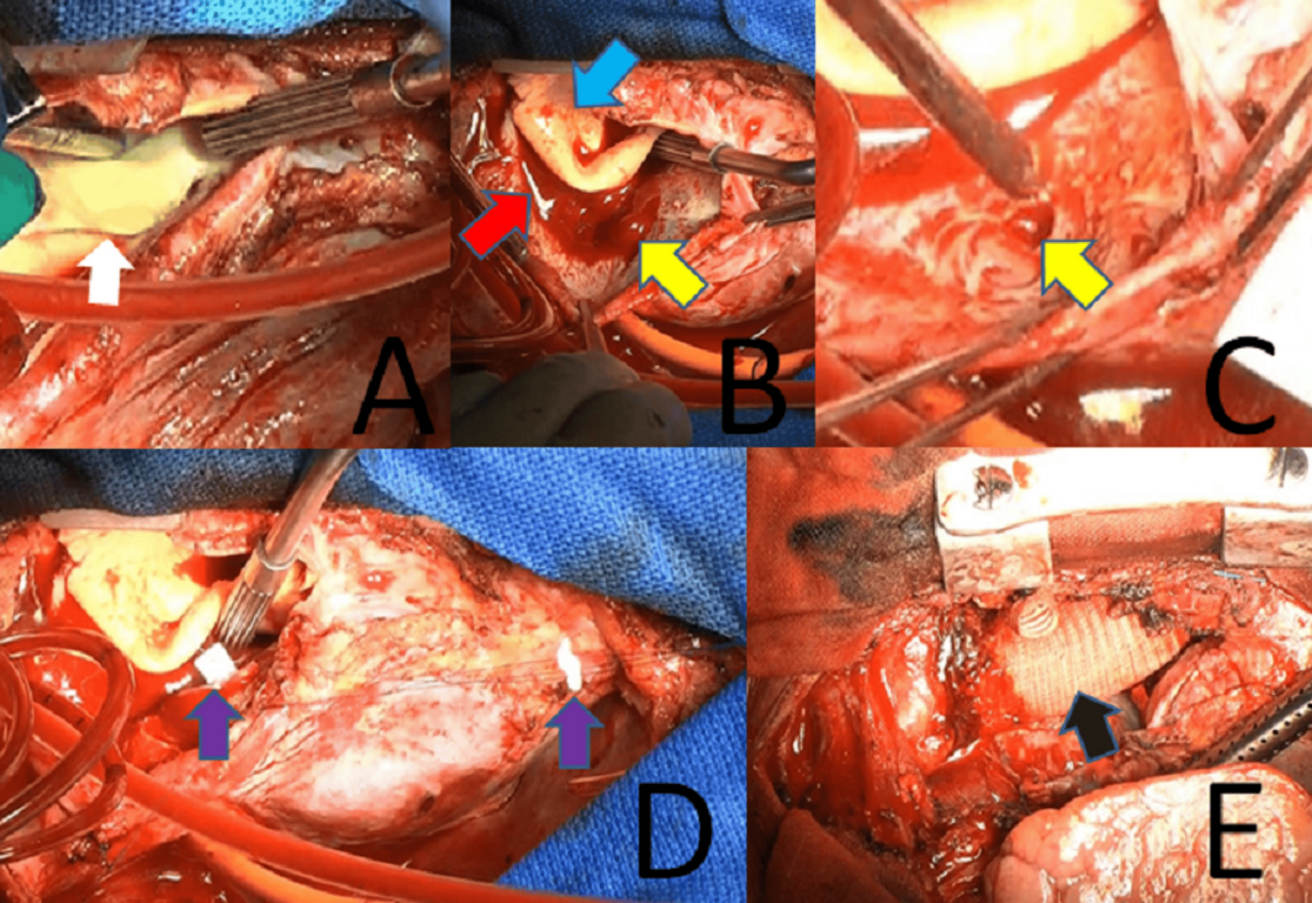
Intraoperative findings A: A 3-cm intimal tear (white arrow) was identified in the healthy aortic wall just behind the previous aortotomy, extending longitudinally from the sinotubular junction B: A fistula (yellow arrow) communicated between the false lumen (red arrow) of the ascending aorta and the right atrium. The blue arrow indicates true lumen. C: The outer layer of the fistula (yellow arrow) was robust. D: The fistula’s outer layer was closed using two 4-0 polypropylene felted sutures (purple arrows) from the false lumen side alone. E: We achieved successful closure of the fistula and central repair by replacement of the ascending aorta (black arrow).

Under general anesthesia, the central venous pressure (CVP) was 26 mmHg and the pulmonary artery pressure was 47/31 mmHg. In case of massive bleeding and subsequent circulatory instability, CPB was established using femoral-femoral bypass, and systemic cooling was initiated. Following median sternotomy, additional drainage and venting tubes were placed in the SVC and right superior pulmonary vein, respectively. Circulatory arrest was achieved when esophageal temperature was 20℃. After transverse and longitudinal incision of the ascending aorta, antegrade selective cerebral perfusion and antegrade cardioplegia were initiated. A 3 cm new intimal tear was identified in the healthy aortic wall just behind the previous aortotomy site, extending longitudinally from the sinotubular junction (STJ) (Figure 3A). An 8-mm fistula was observed from the false lumen of the ascending aorta (Figure 3B). The bioprosthetic aortic valve was intact. Sinuses of Valsalva were dissected, but left and right coronary orifices were free from dissection and intact. Therefore, only the ascending aorta was planned to be replaced. The fistula’s outer layer was so robust (Figure 3C) that it was closed using two 4-0 polypropylene felted mattress sutures only from the false lumen side (Figure 3D). After forming the distal stump, a one-branched J-graft prosthesis (28 mm; Japan Lifeline, Tokyo, Japan) was anastomosed. Total circulatory arrest lasted two hours and 27 minutes, after which systemic circulation was initiated via the graft branch. The proximal stump was formed just above the STJ, and proximal anastomosis was completed. CPB weaning was uneventful. We achieved successful closure of the AAF and central repair of TAAD by replacement of the ascending aorta (Figure 3E). Cardiac arrest time was six hours and 25 minutes and operation time was 10 hours and 54 minutes.

Postoperative course

Postoperatively, the patient was extubated on POD 5 and experienced dysarthria and right hemiparesis. Magnetic resonance imaging revealed a left pontine infarction, which was thought to be due to a scatter of plaque stuck to the aortic wall or thrombus in the false lumen during CPB. Other postoperative examinations were unremarkable, and the patient was transferred to another hospital for rehabilitation on POD 43.

Follow-up

He was discharged from the rehabilitation hospital on POD 158 and visited our hospital on POD 167 for postoperative regular checkup. Contrast-enhanced CT showed that the dissected descending aortic aneurysm had enlarged from 45 mm to 53 mm in diameter over six months. A new re-entry was identified at the origin of the left subclavian artery, in addition to the one at the distal end of the descending aortic dissection. The patient underwent 1-debranching thoracic endovascular aortic repair (TEVAR) for re-entry closure at Kagoshima University Hospital. He has been alive for five years since the TEVAR.

## Discussion

The onset of TAAD in our case was considered the time when the chest and back pain occurred. The severe pain of aortic dissection may cause vagal reflexes, including hypotension and bradycardia [12]. He had no coronary disease, and the sinus bradycardia of 40 beats per minute following the chest and back pain suggested the immediate occurrence of aortic dissection.

Even in the patient diagnosed with TAAD at the former hospital, careful physical examination, including inspection, palpation, and auscultation, and repeated echocardiography are essential. TAAD typically presents as acute chest pain and hemodynamic collapse, while AAFs caused by TAAD after PCS may uniquely present pan systolic murmur, and right and left ventricular overload. A pan-systolic murmur after valve surgery raises concern about postoperative complications, including patient prosthesis mismatch, paravalvular leakage, structural valve degeneration, endocarditis, shunt, and so on. In our case, we suspected AAF during the first auscultation on the admission day. However, initial transthoracic echocardiography failed to reveal a clear shunt flow and repeated examinations eventually identified the shunt. This suggests that early detection of AAF following TAAD, using transthoracic echography, may be diagnostically challenging due to a narrow stream of the shunt or a poor echographic window of the ascending aorta. Transesophageal echography or contrast-enhanced CT with regular contrast dose could have confirmed AAF earlier and led to earlier intervention. Disproportionate dilation of the right atrium on CT can also serve as a diagnostic clue for AAF [13]. When postoperative systolic murmur, right heart failure, and pulmonary edema are present, a left-to-right shunt should be proactively considered and examined earlier.

Considering the surgical indication of the ascending aorta, in our case, preoperative evaluation of the initial DVR revealed that the diameters of the ascending aorta and sinus of Valsalva 45 mm each did not meet Japanese surgical intervention thresholds. According to the 2022 American College of Cardiology and the American Heart Association (ACC/AHA) guidelines for diagnosis and management of aortic disease [14], concomitant ascending aortic replacement is a Class 2a recommendation for experienced surgeons when the maximum diameter of the ascending aorta reaches 45 mm or more. However, the 2020 Japanese guidelines on the management of valvular heart disease [15] and on the diagnosis and treatment of aortic aneurysms and aortic dissections [1] do not stipulate surgical recommendations for ascending aortas less than 50 mm in non-Marfan and tricuspid aortic valve patients. The discrepancy of those guidelines and his ripe old age, 76 years old, partly contributed to our decision to avoid concomitant replacement of the ascending aorta or aortic root at the first valvular operation. When preoperative evaluation of an aortic valve operation reveals an ascending aorta diameter of 45 mm or more, performing the concomitant replacement of the aortic root, ascending aorta, and partial/hemi arch should be carefully discussed for future aortic aneurysm rupture or dissection. For aortic high-risk patients, alternative aortic cannulation strategies, including axillary and femoral artery perfusions, are also options.

While pathological findings of the dissected aortic wall were unavailable in our case, cystic medial necrosis is the most common pathology associated with ascending aortic aneurysms, such as in Marfan and Ehlers-Danlos syndromes [16]. Some cardiovascular surgeons suppose even when the ascending aorta measures less than 50 mm, it is advisable to consider performing concomitant replacement of the ascending aorta or to avoid using the ascending aorta as a cannulation site to mitigate the risk of intraoperative and future dissection. Natsuki et al. [17] went so far as to suggest from a clinical trial that surgery to prevent aortic dissection or enlargement should be considered when the ascending aorta exceeds 40 mm in diameter.

Regarding the surgical strategy of aorto-right atrial fistula after PCS, special considerations are necessary when establishing CPB, especially in the case of heart failure due to left-to-right shunts. High CVP caused by shunt increases the risk of mediastinal organ and lung injuries during a second median sternotomy, leading to massive bleeding and circulatory instability. Therefore, establishing CPB before sternotomy is crucial to achieve circulatory stability [18]. The arterial line of the femoral or axillary artery ensures sufficient systemic perfusion, and the venous line of the femoral vein allows the right atrium and ventricle to collapse completely, reducing the risk of organ injury. Aorto-right atrial fistulas can be repaired from the false lumen side alone, as in our case, from the right atrial side alone, or from both sides. In the both-sides approach, the shunt would be first closed from the atrial side, and then it would be reinforced from the false lumen side with glue or a felt sandwich.

To explore surgical management of aorto-right atrial fistulas caused by TAAD after PCS, we searched PubMed and Google Scholar using the terms “aorto-right atrial fistula,” “dissection,” and “previous /after cardiac surgery.” Seven case reports and eight clinical cases were identified [13,19-24] (Table 2). These fistulas, ranging from 5 to 25 mm in diameter, were reported three or more years after PCSs, including aortic valve replacement (AVR), mitral valve replacement (MVR), coronary artery bypass grafting (CABG), ascending aortic replacement, and heart transplantation. In our case, TAAD occurred only six months after DVR. This is partly because antihypertensive drug, including calcium channel blocker and angiotensinⅡ receptor blocker, had not been administered. A few cases involved multiple fistulas. Interestingly, in some cases including our case, the TAAD entries were not located at sites of previous aortotomy, cannulation, cross-clamping, or vein graft anastomosis, contrary to earlier reports [6-8]. Peri-aortic remodeling and adhesions due to PCS might deprive ascending aorta of original mobility and elasticity, resulting in dissection at intact aortic wall as mechanical complication. Most fistulas were closed using pledgeted or felted sutures. Larger fistulas would require patch closure [23]. In all TAAD cases, the ascending aorta or aortic root was replaced. Regarding specified vital prognosis among previous reports, our patient has been alive for five years, which is the best outcome.

**Table 2:** Cases of aorto-right atrial fistula caused by Type A aortic dissection after previous cardiac surgery Ao: Aorta PCS: Previous cardiac surgery AVR: Aortic valve replacement AR: Aortic valve regurgitation MVR: Mitral valve replacement MR: Mitral valve regurgitation AAF: Aortic atrium fistula CABG: Coronary artery bypass grafting TAAD: Type A aortic dissection LCO: Left coronary artery orifice

Author	Published Year	Age Sex	Diameter of preoperative ascending Ao	PCS	Time between PCS and AAF by TAAD	Character of AAF	Entry of aortic dissection	AAF closure technique	TAAD operation	Outcome
Tayama et al.[19]	1995	58 F	52 mm	AVR for AR	3 years	Multiple small fistulas	1.5 cm distal to the LCO and at the LCO	Pledgeted sutures from the false lumen	Aortic root replacement and reconstruction of the coronary arteries	15 months alive
Scalia et al. [20]	1997	61 F	Unknown	Redo-MVR	7 years	Unknown	Not clearly identified	Oversewing from the right atrium	Replacement of the ascending Ao	One year alive
		76 M	Unknown	CABG	7 years	Unknown	Unknown	Unknown	Replacement of ascending Ao and CABG	12 years alive
Fujii et al. [21]	1998	74 F	Unknown	Replacement of ascending Ao for TAAD	4 years	10mm	Proximal to the graft	4-0 polypropylene sutures from the false lumen	Aortic root replacement using Piehler's method	10 months alive
Caruso et al. [22]	2000	59 F	Unkown	Heart transplantation for dilated cardiomyopathy	6 years	25×6.7 mm	Previous anastomosis of the donor ascending Ao to the recipient one	Unknown	Replacement of the ascending Ao	Death within 48 hours of surgery
Nakano et al. [23]	2000	65 M	Unknown	AVR	15 years	Two neighboring fistulas	Between the previous aortotomy and the aortic annulus	A horizontal mattress suture with two teflon felt strips from the right atrium	Aortic root replacement using Carel's patch method	Alive
Panzarella et al. [24]	2005	73 M	Unknown	AVR	15 years	Unknown	Unknown	4-0 pledgeted polypropylene sutures from the false lumen	Aortic root replacement	Alive
Kang and Hsieh [13]	2016	82 M	Unknown	AVR for AR	9 years	5 mm	Near the previous aortotomy	4-0 polypropylene sutures from the false lumen	Replacement of the aortic valve and ascending Ao	Alive
Our case	2025	76 M	45 mm	AVR for AR and MVR for MR	6 months	8 mm	Just behind the previous aortotomy	Two 4-0 polypropylene felted sutures from the false lumen	Replacement of the ascending Ao	5 years alive

Our case demonstrates the successful surgical management of an aorto-right atrial fistula caused by subacute TAAD six months after DVR and LAA occlusion. While TAAD after PCS is a rare complication, subsequent AAF presents as a lethal condition needing a hemodynamic intervention. Recently, Mammadli et al. [25] and Shimoishi et al. [26] reported successful embolization cases of postoperative AAFs and aortic root pseudoaneurysms using Amplatzer Duct Occluder Ⅱ (St. Jude Medical, Inc., St. Paul, MN, USA) and Amplatzer Vascular Plug Ⅱ (AVPⅡ, Abbott Medical, Santa Clara, CA, USA), respectively. Such devices may offer viable alternatives for managing AAFs in patients with high surgical risks or poor tolerance for surgery. In particular, AVPⅡ, in which the plug diameter ranges from 3 to 22 mm, is repositionable and self-expandable, ensuring precise placement and less migration [27]. Additional cases should be studied to evaluate their efficacy in this context.

## Conclusions

Successful closure of the fistula and replacement of the ascending aorta were achieved in a patient with aorto-right atrial fistula caused by subacute TAAD after DVR and LAA occlusion. When preoperative imaging shows an ascending-aorta diameter ≥45 mm, it may be reasonable to discuss the concomitant replacement of aortic root, ascending aorta and partial/hemi arch, and cannulation strategies, in line with ACC/AHA Class IIa guidance. This suggestion is context-dependent, especially in high-risk or reoperative scenarios. Given the paucity of TAAD after PCS, further multicenter series or registry data are needed to validate the optimal ascending aortic diameter threshold and cannulation approaches for preventing postoperative dissection.
